# The ecological consequences of the timing of extreme climate events

**DOI:** 10.1002/ece3.9661

**Published:** 2023-01-24

**Authors:** Elizeth Cinto Mejía, William C. Wetzel

**Affiliations:** ^1^ Department of Entomology and Ecology, Evolution, and Behavior Program Michigan State University East Lansing Michigan USA; ^2^ Department of Integrative Biology and AgBioResearch Michigan State University East Lansing Michigan USA

**Keywords:** climate change, extreme climatic events, extreme weather events, global change, ontogeny, timing

## Abstract

Climate change is increasing the intensity, duration, and frequency of extreme climate events (ECEs). These ECEs can have major ecological consequences, e.g., changing nutrient flows, causing extirpation, and altering organismal development. Many ECEs are discrete events that occur at distinctive times during the biological processes they impact. Because of this, ECEs are likely to have differing ecological impacts depending on when they happen, yet we lack on studies that explore how the ecological consequences of ECEs vary with when they occur. Drawing upon evidence from physiological, population, and community ecology, and previous work on ecological disturbances, we suggest that the consequences of ECEs will be sensitive to when they occur. We illustrate the importance of timing by showing how the effects of an ECE could vary depending on when it occurs through the course of (1) organismal ontogeny, (2) population dynamics, and (3) community assembly. An enhanced focus on the timing of extreme weather in climate change research will reveal how and when ECEs are altering ecosystems, possible mechanisms behind these impacts, and what ecosystems or species are most vulnerable to ECEs, helping us to make more informed predictions about the ecological consequences of climate change.

## Graphical abstract

Extreme climatic events (ECE) are increasing in intensity, frequency, and duration globally. We present evidence that the ecological consequences of ECEs are highly sensitive when they occur during the progression of biological processes at each of these scales.

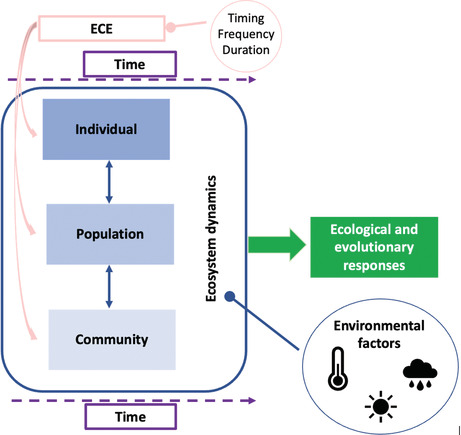

## 1 INTRODUCTION

In the last decade, a growing number of ecological studies have focused on extreme climatic events (ECEs), which are increasing in frequency and severity across the globe (Jentsch et al., 2007; Meehl & Tebaldi, 2004). ECEs, such as heat waves, drought, heavy rainfall, cold snaps, and cyclones, are characterized by short time periods of severe weather that disturb ecological systems (Smith, 2011). Recent work has demonstrated that ECEs can contribute as much to the ecological consequences of climate change as changes in mean climatic conditions (Maxwell et al., 2019), making an understanding of the ecology of ECEs crucial to predicting and responding to the consequences of climate change in general. In this paper, we propose that a key piece missing from our understanding of the ecological consequences of ECEs is the role of their timing—when an ECE occurs relative to the timing of the biological processes it impacts. Resolving the role of timing will help researchers predict the consequences of ECEs and identify how, which, and when systems are most sensitive to the effects of ECEs.

Over the last few decades, an increasing number of climate change experiments have adopted temporally realistic abiotic regimes, including manipulations of the frequency, duration, and amplitude of extremes. For example, manipulating heavy rainfall events after a fire, de Luís et al. (2005) found that extreme rain reduces the survival of plant seedlings. Increased drought period events altered plant phenology delaying flowering in two Mediterranean shrublands (Llorens & Penuelas, 2005). More recent reviews (Jentsch et al., 2007; Thompson et al., 2013) call out the importance of studying climate variability and incorporating extreme values into future climate change research as well as the need to address the timing of such events. Many ECEs, like many disturbances, are discrete events that occur at specific times during the biological processes that they impact (Sergio et al., 2018), and this fact suggests that the consequences of ECEs will depend on the timing of biological and ecological events in relation to the timing of the ECE.

The role of event timing has received significant attention in the ecological disturbance literature, where timing has been found to be of great importance for how disturbances affect organisms, populations, and communities. For example, physical soil disturbance can drive some plant species to extinction depending on when it occurs during community assembly (Crawley, 2004). Similarly, in aquatic systems, algal communities vary in resistance to spates (flooding events) through community dynamics (Peterson & Stevenson, 1992). Studies of climate change, however, have been slow to adopt the temporally explicit perspective needed to examine how the consequences of ECE depend on when they occur. Moreover, the effects of ECE timing may differ in important ways from our understanding of timing from the disturbance literature. First, many extreme climate events differ from most traditionally studied ecological disturbances. For example, events like heat waves, while stressful for many organisms, are not truly destructive (Jentsch et al., 2007). They affect ecology primarily through sublethal effects (Conradie et al., 2019; Domínguez et al., 2021) and may directly benefit some species, e.g., by increasing growth rates of heat‐tolerant and temperature‐limited species. Second, climate‐related events often have characteristic timings, meaning that predicting their consequences requires studying these specific timings (e.g., hurricane season), rather than timing in general. Finally, some extreme climate events can have very large spatial extents, like the 2003 heat wave that affected all of Europe (García‐Herrera et al., 2010), making spatial processes like recolonization potentially less important for ECEs than they are in traditional disturbance dynamics.

**FIGURE 1:**
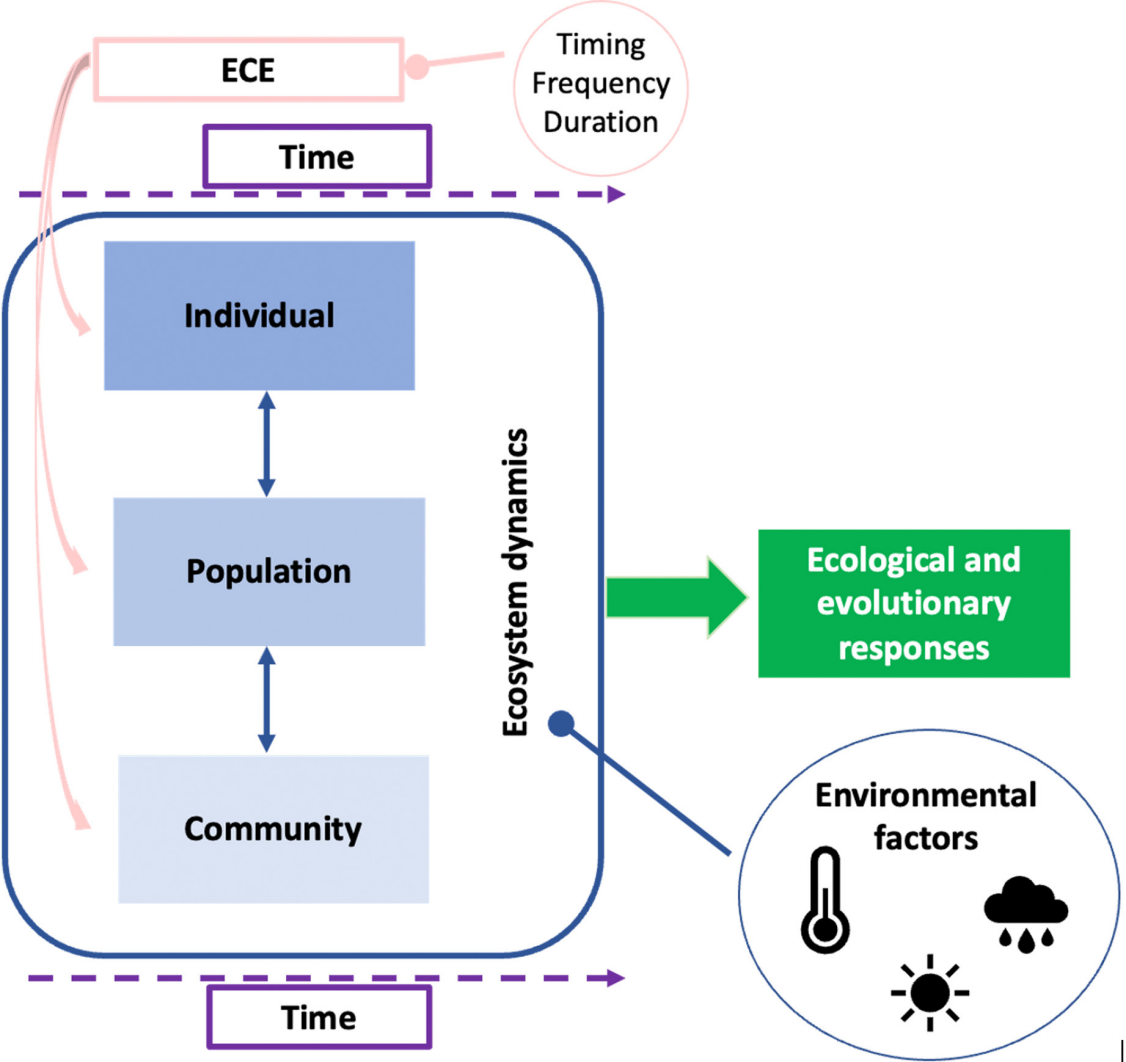
The timing (when the ECE happens), frequency, and duration of an ECE in relation to the timing of events at the individual, population, or community scale will determine future ecological and evolutionary responses, and when we might see those responses based on the ecosystem adaptation to environmental factors.

In this perspective, we explore how the consequences of ECEs might depend on biological timing at three scales (Figure 1): At the individual scale, we explore how the ecological consequences of an ECE will depend on the physiological and ontogenetic stages of organisms when an ECE occurs. At the population scale, we examine how the ecological effects of an ECE are contingent upon when the event occurs through the course of population dynamics. At the community scale, we explore how the ecological effects of an extreme climatic event vary with when an event occurs through the course of community dynamics. Finally, we show how considering differences in the timing of the ecology and biology of individuals, populations, and communities across ecosystems leads to testable predictions about when ecosystems might be susceptible to ECEs and in which ecosystems variation in timing might be more influential. Identifying how the outcomes of an ECE might vary through time and across ecosystems will improve our ability to understand the impacts of extreme weather. Throughout our paper, we use the term timing to mean when an ECE happens, in contrast to studies that focus on duration, frequency, or other aspects of timing.

## 2 INDIVIDUAL‐LEVEL TIMING

**FIGURE 2:**
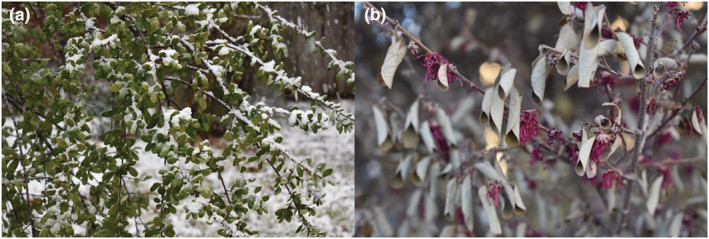
Effects of ECEs at different timings during species ontogeny. A Persimmons species (*Diospyros* sp.) during vegetative stages (a) and a Chinese redbud (*Cersis chinensis*) during flowering stages (b) both damaged after the 2021 cold wave in Texas, USA. Photo credit: Keri Greig.

Organisms undergo biological changes through time including continuous changes in traits through growth and development and discrete changes during life history events (e.g., breeding or migration). In this section, we discuss how the ecological consequences of an ECE will depend on the current ontogenetic stages and states of the organisms present in the community when and where the ECE occurs (Figure 2). Extreme conditions alter physiological processes, and these effects depend on when the event happens during an organism's ontogeny. Throughout the paper, we define susceptibility or vulnerability of an organism to an ECE as the period or periods of time during organism life that due to physiological changes an ECE has a higher chance of altering the organism's response to the ECE. The susceptibility or vulnerability of an organism to an ECE is the extent to which its biological processes are perturbed by an ECE at a given point in time. For example, when considering extreme heat temperatures, all insect stages present at the time of the event will experience extreme temperatures, but the growth and survival consequences for each individual will depend on its ontogenetic stage. This stage‐specific thermal response (Ma et al., 2021) governs the ontogenetic timing of susceptibility or vulnerability to the stress associated with an ECE.

**FIGURE 3:**
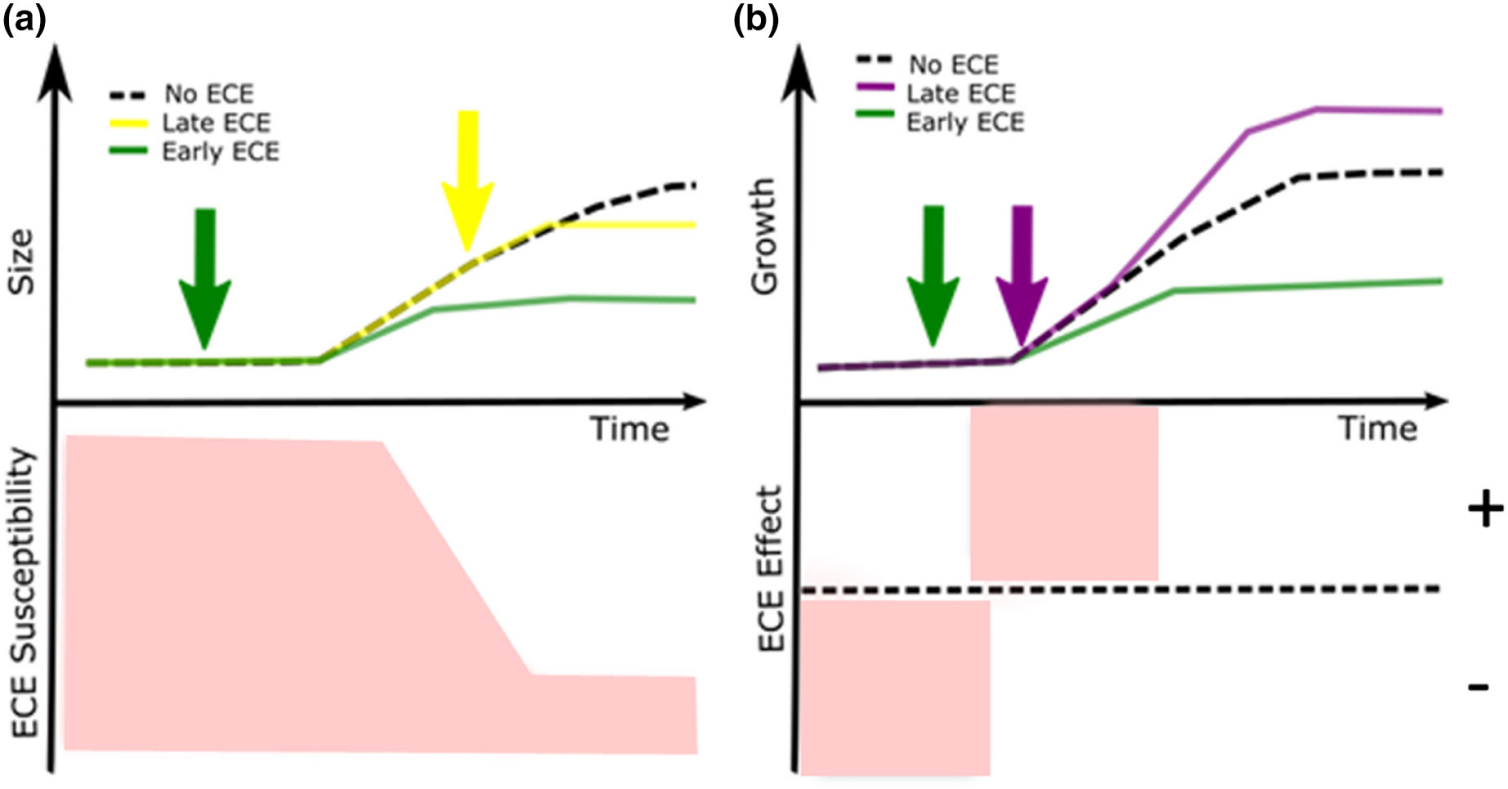
Hypothetical outcomes of an organism's growth change depending on when an ECE happens. (a). Magnitude of the effect: An ECE happening at the early stages of growth (green arrow), when an organism's susceptibility to an ECE is high, the ECE could alter growth rate and total growth for that year. An ECE happening during the later stages of an organism (yellow arrow), when ECE susceptibility is low, might have no effects on total growth or growth rates. (b). Direction of the effect: An ECE happening when the organism's susceptibility to an ECE is high ECE, the ECE could have negative effects on growth (green arrow) due to a negative response, or positive effects on growth (purple arrow) due to a positive response. In both panels, time can be represented in different units, and it is relative to the ontogeny and phenology of organisms.

Evidence from the literature suggests that the timing of ECEs predicts the physiological changes and mortality they cause. For example, the effects of heat waves on the survival of green peach aphids (*Myzus persicae*) depend on aphid age (Gillespie et al., 2012). Crucially this is also true for the nonlethal effects of an ECE on organismal performance. When cereal aphids (*Metopolophium dirhodum*) experience heat events their lifetime fecundity and longevity depend on their life stage during the event (Ma et al., 2004). Similarly, heat waves reduce the growth and development of tobacco hornworms (*Manduca sexta*) if the event happens during the early stages of development but not later in ontogeny (Kingsolver et al., 2021). Even the direction of a response can vary with ECE timing. In both big bluestem (*Andropogon gerardii*) and goldenrod (*Solidago canadensis*), high heat events have opposite effects on photosynthetic rates and productivity depending on the plant stage (Wang et al., 2016). In the case of marine invertebrates, Pandori and Sorte (2019) published a meta‐analysis of over 250 experiments showing that while all life stages are affected by ECEs, younger stages like embryos and larvae are more sensitive to extreme heat. The bottom line is that ontogenetic variation in susceptibility to the stresses associated with ECEs can lead to slight variation in the timing of an ECE to result in ecological consequences that differ quantitatively as well as qualitatively (Figure 3).

A key aspect of how ECE timing will interact with ontogenetic timing is the temporal pattern of trait change through ontogeny. Many traits change gradually and linearly with growth, while other traits, such as life history events, vary nonlinearly or cyclically through time (Post et al., 2001). The relationship between ontogeny and other physiological changes will vary with each trait and should be considered when examining the consequences of an ECE during growth. In marine species, such as mollusks, tolerance to salinity varies nonlinearly through development (Mann & Harding, 2003). Plants like narrow‐leaved plantain (*Plantago lanceolata*) have a positive relationship between age and chemical defense concentrations, and a negative relationship between age and water and nitrogen concentration (Quintero & Bowers, 2018). For example, in the hypothetical case that an ECE caused herbivores to increase consumption rates or plants to produce lower defenses due to environmental stress like heat, this might have a higher impact on younger plantain plants than older ones. If we step back from the plantain example, we can hypothesize that traits that change over time can determine the effects of an ECE. We can use these relationships between traits and growth to predict when and how organisms can be susceptible to ECEs. For example, a heat event experienced by adult diamondback moths (*Plutella xylostella*) led to a 21% decrease in the number of hatched eggs produced by females with resulting population changes (Wei Zhang et al., 2013). These examples and the broader literature suggest that the timing of an ECE is likely more important for organisms that undergo rapid, discrete changes, like metamorphosis in holometabolous insects, than for organisms, like vertebrates, which change more gradually. The former organisms therefore may be more likely to pass the effects of an ECE on to community scales indirectly via species interactions (Table 1).

**TABLE 1:** Example of some testable predictions based on the timing of ECEs.

Hypotheses	Possible predictions
Based on physiological traits, organisms whose susceptibility to an ECE is variable have different responses to the ECE depending on when the ECE happens. *Ex. Organism's tolerance to cold event changing over time* 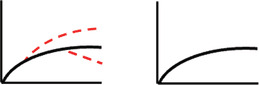	The timing of the ECE will be important and the ECE will influence the organisms' phenology, physiology, behavior, or mortality if susceptibility is low at times. The timing of the ECE will not be important and there will not be a response from the organisms' phenology, physiology, behavior, or mortality if susceptibility is constant over time.
For organisms highly synchronized with the environment, the timing of an ECE can determine the effects of the ECE depending on when the ECE happens. 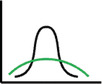	The effects of the timing of the ECE will depend on when the ECE happens for highly synchronized organisms. The effects of the timing of the ECE will not matter for organisms not synchronized with the environment.
In cyclical populations, an ECE can have different effects even at the same population size depending on if the ECE happens as the population is growing vs when the population is declining. 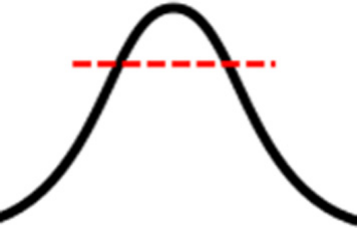	An ECE happening at N = x as the population increases might not have as severe effects as an ECE happening as the population decreases due to population recovery and replacement.

We hypothesize that the timing of how ECE effects are transmitted to higher ecological levels will be dominated by the ontogenetic timing of organisms influential in species interactions—keystone species, foundational species, and ecosystem engineers. Paine (1966) showed how the removal of a main predator, the sea star *Pisaster ochraceus*, increased the diversity in the area by releasing mussels from predation, a foundational species that provides habitat for other species. Pike and Stiner (2007) observed that the timing of a cyclone can be detrimental to turtle populations if the cyclone happens when abundance of young turtles is high due to the mortality of these young individuals. We can expect that an ECE that occurs when a keystone species is vulnerable could lead to high mortality, and this mortality could indirectly lead to changes in species diversity. For example, sea urchins can be voracious grazers with major effects on community composition. Sea urchins can have positive effects by keeping the algae populations low in coral reefs or have negative effects by acting as grazers in an ecosystem (Cabanillas Terán, 2009). A sea urchin (*Diadema africanum*) in Madeira, Portugal, was able to recover quickly from a mass‐mortality event in part because it occurred immediately after the urchin's spawning period (egg‐releasing period). While adults died in mass, the larvae were resistant and contributed to a rapid population recovery (Gizzi et al., 2020).

## 3 POPULATION‐LEVEL TIMING

We suggest that, in addition to depending on ontogenetic timing, the ecological effects of an ECE will depend upon when the event occurs through the course of population dynamics. Moreover, the role and importance of the timing of an ECE will matter differently for populations that follow different characteristic dynamical patterns: relatively steady with little change over time, cycles where abundance varies regularly through time, and chaotic or stochastic with unpredictable change through time (Tuljapurkar, 2013). Additionally, age or stage distributions and genotypic frequencies change through time, and this should structure temporal variation in the consequences of ECEs.

**FIGURE 4:**
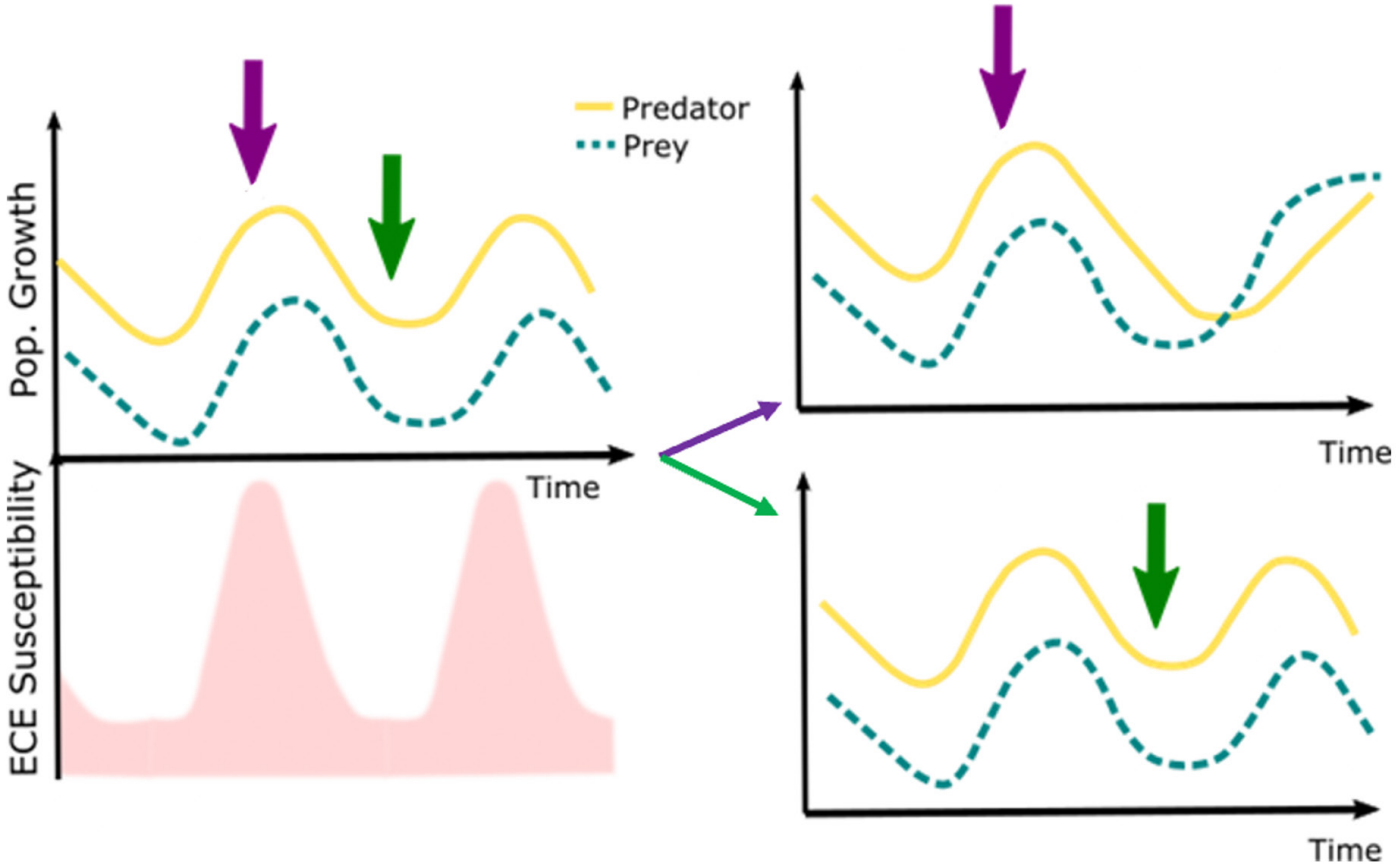
Using dynamic models, Commander & White, 2020 showed the effects of various disturbances based on different predator–prey population dynamics, we hypothesized predator–prey dynamics over time after an ECE. An ECE near or at the peak of growth in populations of predator and prey (purple arrow) could result in a decrease in the predator's population growth and an increase in prey's population growth. An ECE happening near or at the valley of growth in populations of predator and prey (green arrow) could result in no changes in predator–prey dynamics.

Based on previous studies, the susceptibility to varying ECEs and the rates of growth, mortality, and behavior of individuals changing over time will drive population‐level responses. Climate and ecological data suggest that the ecological effects of a disturbance can range from zero to strongly negative depending on when the disturbance happens. Observational climate change data have shown that a cold wave before a population peak can reduce the population size of a Lepidoptera species (Wagenhoff & Veit, 2011). By experimentally altering rain events on terrestrial ecosystems, Levine et al. (2011) found that the timing of the rain determines population dynamic patterns. Theoretical data also indicate that the consequences of altering a population depend on the life stage of the population suggesting that population vulnerability is strongly related to population phenology (Coulaud et al., 2013). For example, mathematical models found that stochastic disturbances can alter seed bank populations (Eager et al., 2013), and the seasonal timing of animal harvesting is a major predictor of the recovery and subsequent transient population dynamics (Angulo & Villafuerte, 2004). After drought events during the breeding season, the Southern pied babbler (*Turdoides bicolor*) recovered their population size a year after the event through compensatory breeding (Bourne et al., 2020). For the Southern pied babbler, long‐term effects might depend on how many individuals remain after the ECEs to start the population recovery and how drought events affect different ages. For populations where the young are more affected than adults (increasing young mortality) by an ECE and that experience an ECE before or during the population peak, we hypothesize that the population will recover within the same breeding season or the year after due to a compensatory breeding response (Figure 4). While this hypothesis is within one or two breeding seasons (short time scale), the effects of an ECE can have different outcomes depending on the reproductive value of the individuals remaining (long time scale). Individuals with high reproductive value like second‐year individuals (Benítez Joubert & Tremblay, 2003) can help the population grow long term (Sæther et al., 2007). If the ECE causes high mortality at a time when the individuals with high reproductive value are affected by the ECE, the population might not be able to recover.

The timing of an ECE will be important when considering the consequences of extreme weather on populations that cycle with another species, such as in predator–prey dynamics. In a predator–prey scenario, we may observe that the ECE will first alter the dynamics of the prey or the predator, with the magnitude of the effects depending on the timing of the populations, later cascading to consequences on predator–prey dynamics (Commander & White, 2020) (Figure 4). Finally, in a population that has non‐stationary dynamics we could observe various scenarios where the timing of ECE can have unpredictable effects or no effects. Alternatively, an ECE could act as a selection pressure depending on when it happens relative to population dynamics involving temporal variation in genotypic frequencies and eco‐evolutionary dynamics. Campbell‐Staton et al. (2017) found that extreme winter storms can shift eco‐evolutionary dynamics in anole lizards, indicating that a one‐time ECE event can alter evolutionary patterns and suggesting that the consequences of the ECE itself may depend on the timing of eco‐evolutionary dynamics. Fruit flies (*Drosophila melanogaster*) show rapid phenotypic adaptation over short time scales (Bergland et al., 2014). Not many experiments explore the importance of manipulating the timing of an ECE along population dynamics, although previous studies have indicated the importance of considering the timing of ECEs.

## 4 COMMUNITY‐LEVEL TIMING

**FIGURE 5:**
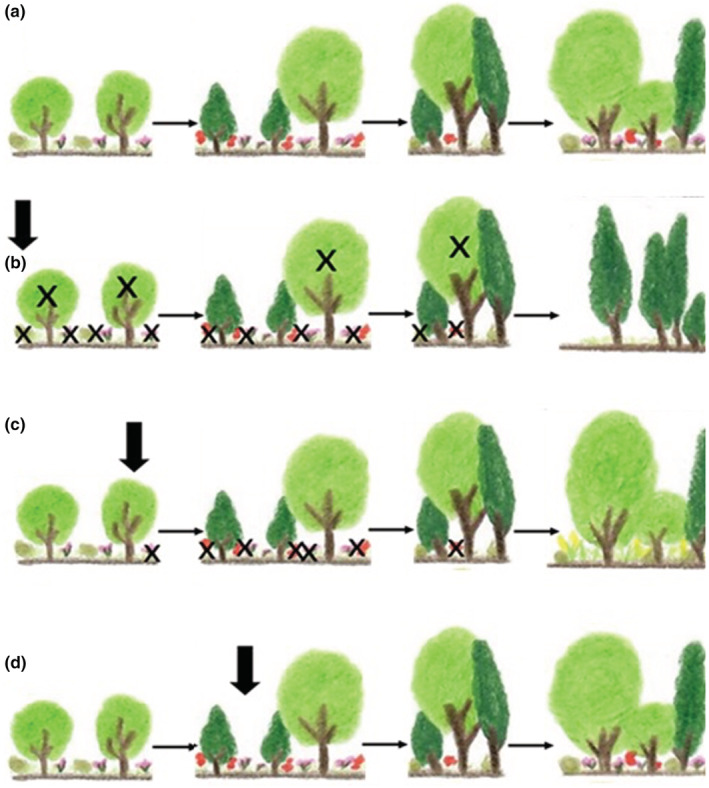
Building upon the work by Suarez & Kitzberger, 2008 and Anderegg et al., 2013 where due to mortality of certain species an ECE can shift the species composition in a forest and have indirect effects on habitat structure and quality, we represent this hypothetical community assembly over time. (a) There is no ECE, and the community assembly develops under the expected trajectory. (b) An ECE (black vertical arrow) that happens during the early stages of the community assembly increases the mortality of understory key species (purple, red, and green), and tree species. The community becomes a different type of ecosystem with one species dominating the landscape and a decrease in diversity. (c) An ECE that happens during mid‐early stages of the community assembly increases the mortality of most species (purple and red). (d) An ECE that happens during mid‐stages of the community assembly has no effects on the community and we do not observe high impacts on diversity and structure due to the ECE.

Finally, we propose that the ecological effects of an ECE on communities are also contingent upon when the event occurs along the time scale of community dynamics. Based on past studies, the effects of the variation in ECE timing will differ across communities that have different temporal patterns of community dynamics. For example, in the disturbance literature researchers have shown that a single disturbance can alter species composition and community assemblies (Collins et al., 2017; Jauni et al., 2015) and that based on the trajectory of the community, the timing of a disturbance can determine what species can dominate a community (Smith, 2006; Turner et al., 1997). Communities can be stable at an equilibrium, changing through succession or assembly, exhibiting patch dynamics, or simply varying unpredictably. We hypothesize that the timing of an ECE is less relevant for a stable community that changes slowly through time, such as a community in the late stages of assembly. Of course, even if the timing of community dynamics is relatively unimportant for a given community, the timing of individual ontogeny and population dynamics within that community should still matter for the consequences of an ECE (Figure 5).

Communities can experience changes through time in abundance, diversity, species composition, trophic structure, and species interactions (Dornelas et al., 2018; Ushio et al., 2018), and we hypothesize that the effects of an ECE depend on the levels of these features when the event occurs. For example, tree species diversity can mitigate the impacts of a disturbance on the carbon cycle (Pedro et al., 2015). Based on this study, diversity and resilience have a positive relationship where diverse ecosystems experience lower disturbance‐induced variability in the ecosystem's trajectory. The synchrony between a population's phenology and seasonality will also be important when considering community dynamics. For example, flooding at different times can have no impact on some plant species that are not affected by seasonality but can alter recolonization of other species if the flooding happens late in the season (Barrat‐Segretain & Bornette, 2000). Species' roles in a community and when these species are part of the community can serve as a predictor of when the timing of an ECE will be important. In an ecosystem that experiences variation in species diversity through time like undergoing assembly processes, we would expect windows of time (e.g., stages of low diversity) when the ECE could have higher or lower impact vs a community where species diversity is more constant.

In addition, when an ECE happens during the course of metacommunity dynamics the timing of ECEs will matter. An ECE that happens during patch extinction or when colonization among patches is high could have less consequences on the dynamics of the metacommunity compared to when patches are experiencing higher replacement. For example, the higher dispersal could mitigate the immediate effects of an ECE that only affects a certain area.

## 5 FOCUSING ON THE TIMING OF BIOLOGY LEADS TO NOVEL PREDICTIONS ABOUT THE EFFECTS OF ECES ACROSS SYSTEMS

Above we explored how the timing of an ECE through the progression of organismal ontogeny, population dynamics, and community assembly may influence the consequences of the ECE. In this section, we show how considering biological timing, and differences in biological timing across systems, may lead to novel insights and hypotheses about the ecology of ECEs and the vulnerability of different systems to ECEs. We illustrate how a temporally explicit approach to ECEs advances our thinking by focusing on two aspects of timing: biological synchrony and the magnitude of change in an ecosystem across time.

A major difference in biological timing across ecosystems is the level of synchrony across scales of organization (Wang et al., 2019). We predict that ecosystems with phenologies highly synchronized with the environment will be more sensitive to the timing of an ECE, and that the duration and frequency of synchronized events will interact with this ECE timing. For example, the relatively high synchrony of germination and flowering in alpine plant communities with the environment (Hall et al., 2018) could lead to a strong relationship between the timing of an ECE and the strength of its effects in these systems, with strong effects during germination and flowering, which tend to be sensitive stages for many plants. In contrast, the asynchrony present in tropical plant communities could lead ECE effects to average out through time, reducing the sensitivity of the system to ECE timing. While the ECE might have high impacts on a tropical community, the timing of the ECE is likely to be less important.

Another component of biological timing is the amount of change systems exhibit within a year. Based on elevation and latitude, some ecosystems will be more variable than others in moisture, temperature, and other environmental factors. For example, in South America, the northern inner tropics receive a consistent amount of water throughout the year while in outer tropics precipitation can vary by over 90% between seasons (Sagredo et al., 2014). In a location with low weather variability, we would see less change in response to the weather, and the timing of an ECE will be less important compared to locations of high weather variability. These changes can be measured as total biomass, fitness and community metrics, or ecosystem services. In the case of a location with high weather variability, we present two possible hypotheses on how ecosystems can respond to the timing of an ECE. First, the timing of an ECE could be more important in systems that experience more change due to weather variability simply because responses to events at different times may differ more when underlying conditions also differ more through time. For example, in a high‐elevation desert with high variability in the timing and amount of rain in a year, like the sagebrush steppe in western North America (Hardegree et al., 2016), heat events could be increasingly damaging with time since the last rain event. Second, and alternatively, the timing of an ECE could be less important in systems that experience more change because organisms and interactions in those systems may be more resilient to extremes in general and might have more capacity for plasticity (Climate Variability Hypothesis) (Molina‐Montenegro & Naya, 2012). For example, in the same sagebrush ecosystem, it is possible that organisms have ontogenetic trajectories that make them increasingly resilient to heat events with time since the last rain event. Although moisture decreases over time, resilience compensates for the possible damage, thereby actually reducing the importance of timing.

Our approach of including the timing on ECEs on future studies has limitations. First, we might not see the effects of ECEs timing until well after the event, and such lagged effects can complicate the interpretation of results. Second, every ECE happens at a certain time of growing season, species ontogeny, and year, making it difficult to divorce each variable. A way to solve the last pitfall would be with factorial experiments that include all the possible scenarios, although we acknowledge the challenge of carrying out these experiments. Although ECEs were not incorporated into their study, Yang et al. (2020) is a great example of experimental design that includes the ontogeny of an herbivore and plants to examine the survival and growth of herbivores, as well as Kharouba and Yang (2021) examining direct and indirect warming effects on interacting species.

## 6 CONCLUSION

The fact that the effects of ECEs are a major part of the overall ecological consequences of climate change indicates that resolving the impacts of ECEs on ecology will be essential for understanding and responding to climate change. We have argued that understanding ECEs will require ecologists to study ECE timing and adopt a temporally explicit approach that considers not just the frequency and duration of events but also when the events occur relative to the biology of the systems they impact. Making this approach a reality will require empirical data and experiments that compare the effects of ECEs that happen at different times through biological scales.

Combining the timing of ECEs and the timing of species, populations, and communities into future analysis will increase the accuracy of our predictions on their short‐ and long‐term consequences, identify previously overlooked groups of organisms and regions that are susceptible to ECEs, inform policy, and enable management recommendations that promote ecological resiliency to the new reality of ECEs.

## AUTHOR CONTRIBUTIONS

**Elizeth Cinto Mejía:** Conceptualization (equal); investigation (lead); methodology (equal); project administration (equal); resources (lead); writing – original draft (lead); writing – review and editing (equal). **William C. Wetzel:** Conceptualization (equal); methodology (equal); project administration (equal); supervision (lead); validation (lead); writing – review and editing (lead).

## CONFLICT OF INTEREST

Both authors declare no conflict of interest.

## FUNDING INFORMATION

Funding was provided by the Michigan State University T. Wayne and Kathryn Porter Graduate Fellowship Endowment Fund., Ecology, Summer fellowship in Evolution, and Behavior, and Summer Critical Needs fellowship.

## Data Availability

Data sharing not applicable to this article as no datasets were generated or analysed during the current study
